# Imaging-based outcome prediction in posterior circulation stroke

**DOI:** 10.1007/s00415-022-11010-4

**Published:** 2022-03-07

**Authors:** Helge C. Kniep, Sarah Elsayed, Jawed Nawabi, Gabriel Broocks, Lukas Meyer, Matthias Bechstein, Noel Van Horn, Marios Psychogios, Götz Thomalla, Fabian Flottmann, André Kemmling, Susanne Gellißen, Jens Fiehler, Peter B. Sporns, Uta Hanning

**Affiliations:** 1 Department of Diagnostic and Interventional Neuroradiology, University Medical Center Hamburg-Eppendorf, Hamburg, Germany; 2 Department of Radiology, Charité School of Medicine and University Hospital Berlin, Berlin, Germany; 3 Department of Neuroradiology, University Medical Center Marburg, Marburg, Germany; 4 Department of Diagnostic and Interventional Neuroradiology, Clinic for Radiology and Nuclear Medicine, University Hospital Basel, Basel, Switzerland; 5 Department of Neurology, University Medical Center Hamburg-Eppendorf, Hamburg, Germany

**Keywords:** Posterior circulation stroke, Outcome prediction, Machine learning, Pc-ASPECTS

## Abstract

**Background and purpose:**

We developed a machine learning model to allow early functional outcome prediction for patients presenting with posterior circulation (pc)-stroke based on CT-imaging and clinical data at admission. The proposed algorithm utilizes quantitative information from automated multidimensional assessments of posterior circulation Acute Stroke Prognosis Early CT-Score (pc-ASPECTS) regions. Discriminatory power was compared to predictions based on conventional pc-ASPECTS ratings.

**Methods:**

We retrospectively analyzed non-contrast CTs and clinical data of 172 pc-stroke patients. 90 days outcome was dichotomized into good and poor using modified Rankin Scale (mRS) cut-offs. Predictive performance was assessed for outcome differentiation at mRS 2, 3, 4 and survival prediction (mRS ≤ 5) using random forest algorithms. Results were compared to conventional pc-ASPECTS and clinical parameters. Models were evaluated in a nested fivefold cross-validation approach.

**Results:**

Receiver operating characteristic areas under the curves (ROC-AUCs) of the test sets using conventionally rated pc-ASPECTS reached 0.63 for mRS ≤ 4 to 0.68 for mRS ≤ 5 and 0.73 for mRS ≤ 5 to 0.85 for mRS ≤ 2 if clinical data were considered. Pure imaging-based machine learning classifier ROC-AUCs were lowest for mRS ≤ 4 (0.81) and highest for mRS ≤ 5 (0.87). The combined clinical data and machine learning-based model had the highest predictive performance with ROC-AUCs reaching 0.90 for mRS ≤ 2.

**Conclusion:**

Machine learning-based evaluation of pc-ASPECTS regions predicts functional outcome of pc-stroke patients with higher accuracy than conventional assessments. This could optimize triage for additional diagnostics and allocation of best possible medical care and might allow required arrangements of the social environment at an early point of time.

## Introduction

Posterior circulation (pc) strokes are frequently associated with poor outcome [8, 17]. Recently reported results from the Basilar artery international cooperation study (BASICS) indicate that functional outcome may still, to a large extent, be dependent on the initial clinical presentation and imaging findings, and, to a lesser extent, on the specific therapeutic strategies [14, 32]. Predicting functional outcome based on the initial clinical and imaging findings might therefore (1) allow for a prognosis of the patient’s long-term functional status (2) optimize triage for additional MR imaging diagnostics and allocation of best possible medical care [36] and (3) facilitate required adaptations in the patient’s social environment including arrangements of long-term care at an early point of time.

Binary quantification of early ischemic changes using the posterior circulation Acute Stroke Prognosis Early CT Score (pc-ASPECTS) was shown to predict functional outcome in patients with suspected pc ischemia [27, 30]. However, conventional binary classifications of pc-ASPECTS regions do not consider all information available from the imaging data: prognostic value carried by changes in texture and small shifts of grey level distributions remains unused. The accuracy of conventional pc-ASPECTS ratings is also affected by the limited sensitivity of the human eye for subtle early ischemic changes. Moreover, visual assessments of non-contrast CT (NCCT) images suffers from inter- and intra-reader variability and are often interfered by beam-hardening artifacts in the posterior fossa [9, 27, 33].

The integration of clinical data, mainly the baseline National Institute of Health Stroke Scale (NIHSS) was shown to improve discriminatory power [16]. However, although being the most widely used scoring system in patients with acute ischemic stroke, NIHSS has weaknesses when applied to pc strokes partly because deficits such as truncal ataxia, dysphagia and diplopia—that are typical for pc strokes—are not assessed. This explains why patients with pc stroke can have a high probability of an unfavorable outcome at 90 days despite relatively low NIHSS scores at admission [31] and underlines the need of a combined approach of imaging evaluation and clinical scoring [16].

We therefore propose a machine learning (ML)-based evaluation of multidimensional quantitative image features from pc-ASPECTS regions in admission NCCTs combined with clinical data to predict functional outcomes in patients with acute pc strokes.

## Materials and methods

The anonymized data used for training and validation of algorithms that support the findings of this study are available from the corresponding author upon reasonable request.

This multi-center retrospective study was approved by the Ethics Committee of the University of Hamburg and the Hamburg Chamber of Physicians, Hamburg, Germany, and the Ethics Committee of the University of Muenster and the Westfalian Chamber of Physicians, Muenster, Germany, and written informed consent was waived by the institutional review boards. All study protocols and procedures were conducted in accordance with the Declaration of Helsinki.

### Patient characteristics

The study cohort includes consecutive patients with suspected posterior circulation ischemia admitted between April 1, 2010, and February 28, 2019 at two tertiary care stroke centers. Inclusion criteria for this study were (1) documented occlusion of the basilar or intracranial vertebral artery; (2) NCCT performed on admission within 6 h of symptom onset; (3) availability of modified Rankin Scale (mRS) after 90 days (mRS90). Patients were excluded in case of poor imaging quality (artifacts from movement and implants). In total, 172 patients met the inclusion criteria and were selected for the imaging-based analysis. Complete clinical data including NIHSS at admission were available for 149 patients that were selected for all models employing clinical data at admission.

### Image acquisition

NCCT scans with head images obtained from the vertex to the skull base were acquired on a 128-slice dual-source CT scanner (Somatom Definition Flash; Siemens Healthcare GmbH) with tube voltage 120 kV, tube current 340 mA, 5.0 mm slice reconstruction, < 0.5 mm in-plane resolution, as well as on an iCT 256™ scanner (Philips Healthcare, Best, The Netherlands) with tube voltage 120 kV, tube current 300 mA, 4.0 mm slice reconstruction and < 0.5 mm in-plane resolution.

### Visual pc-ASPECTS rating

For all admission NCCT scans, pc-ASPECTS was conventionally assessed by two Neuroradiologists in a consensus rating approach (UH, PS: 8 years of clinical experience in diagnostic neuroradiology in acute care full-service hospitals). pc-ASPECTS allots the posterior circulation 10 points. One point each is subtracted for early ischemic changes on NCCT in left or right thalamus, cerebellum, or posterior cerebral artery territory, respectively, and two points each for early ischemic changes in any part of the midbrain or pons. A pc-ASPECTS score of 10 indicates absence of visible posterior circulation ischemia, a score of 0 indicates early ischemic changes in all pc-ASPECTS territories [7, 27].

### Image pre-processing and pc-ASPECTS feature extraction

To (1) extract information from standardized pc-ASPECTS maps and (2) reduce potential bias in quantitative texture analysis, all NCCT images were registered to a custom MNI (Montreal Neurological Institute)-152 CT reference atlas [10] using two-step affine algorithms. Registration success was visually verified by two Neuroradiologists (UH, PS). Standardized pc-ASPECTS area maps (thalamus left/right (l/r), pons, midbrain, territory of the posterior cerebral artery (PCA) l/r, cerebellum l/r) were derived as follows: First, an experienced Neuroradiologist (UH) performed manual segmentations of the respective regions on the original NCCT images of 63 healthy subjects using Analyze 11.0 Software (Biomedical Imaging Resource, Mayo Clinic, Rochester, MN) [3]. Second, manual segmentations were transformed into standard space by utilizing transformation matrices obtained from image registration to the custom MNI-152 CT reference atlas [10]. Third, all segmentations were added and final standard maps were defined by applying 50% cut-off points.

Quantitative image features were extracted using the PyRadiomics Python package v2.1.0 [35], proposed default settings were used for the analysis. Extracted features comprised 252 first-order features (18 based on unfiltered images, 144 wavelet decompositions, 90 log-sigma Laplacian of Gaussian filtered images) and 966 texture features (82 based on unfiltered images, 544 wavelet decompositions, 340 log-sigma Laplacian of Gaussian filtered images). In total, 1218 quantitative image features were extracted from each of the 1376 included pc-ASPECTS areas.

### Statistical analysis

Univariate logistic regression analysis was conducted based on the entire dataset to investigate conventional odds ratios of the clinical predictors (NIHSS at admission, pc-ASPECTS and age) for good outcome (mRS90 ≤ 2). Using fivefold cross validation, univariate (conventional pc-ASPECTS ratings) and multivariate logistic regression models (conventional pc-ASPECTS ratings, NIHSS at admission and age) were trained to predict functional outcome at dichotomized mRS90 levels of ≤ 2, ≤ 3, ≤ 4 and ≤ 5 (survival).

Imaging-based machine learning prediction of dichotomized mRS90 levels was evaluated using Random Forest algorithms (Python scikit-learn environment v0.20.3 [24]) in a fivefold nested cross validation approach [15]. Random forest classifiers have a comparably low tendency to overfit [4] and support classification tasks also for data sets comprising numerous and heterogeneous predictors. For each study patient, quantitative image features of the eight pc-ASPECTS regions were evaluated for their ability to predict functional outcome (9744 image feature in total per patient). Hyperparameter tuning of the random forest classifiers (total number of features, number of trees, maximum depth of the tree, minimum number of samples to split an internal node, number of features considered for splitting (*m*_try_), minimum number of samples at leaf node) was conducted using grid search algorithms on each training data set within the nested cross-validation layers. Parameters at initiation were set to scikit-learn default values. Selection of features with the highest predictive value was conducted separately for each training data set of the fivefold cross-validation sample split according to Gini impurity measures [18]. For the integrated model, predicted probabilities for good outcome of the logistic regression model using clinical data and of the imaging-based machine learning classifier were averaged.

Receiver operating characteristic (ROC) curves were used to determine the optimal cut-off values according to Youden’s index. For predictive models, ROC curves were generated from results of all cross-validation sets. Confidence intervals (CI) for sensitivities and specificities were bootstrapped (2000 replicates, pROC v1.15 R-package [29]). Bonferroni adjustments were applied to control for alpha error inflation. Furthermore, the classifiers were analyzed using sensitivity, specificity, accuracy, maximum Youden Index, positive predictive value, negative predictive value (ThresholdROC v2.8 R-package) and Matthews correlation coefficient (MCC) [20] metrics (psychometric v.2.2. R-package). MCC evaluates all fields of the confusion matrix and is considered as a favorable measure for unbiased comparisons of binary classifiers [25]. Due to the relatively low class imbalance for all mRS90 cut-off values (event rates for mRS90 ≤ 2: 33%; ≤ 3: 40%; ≤ 4: 56%; ≤ 5: 74%), no additional data augmentation for reducing bias from class imbalance was performed.

**Fig. 1:**
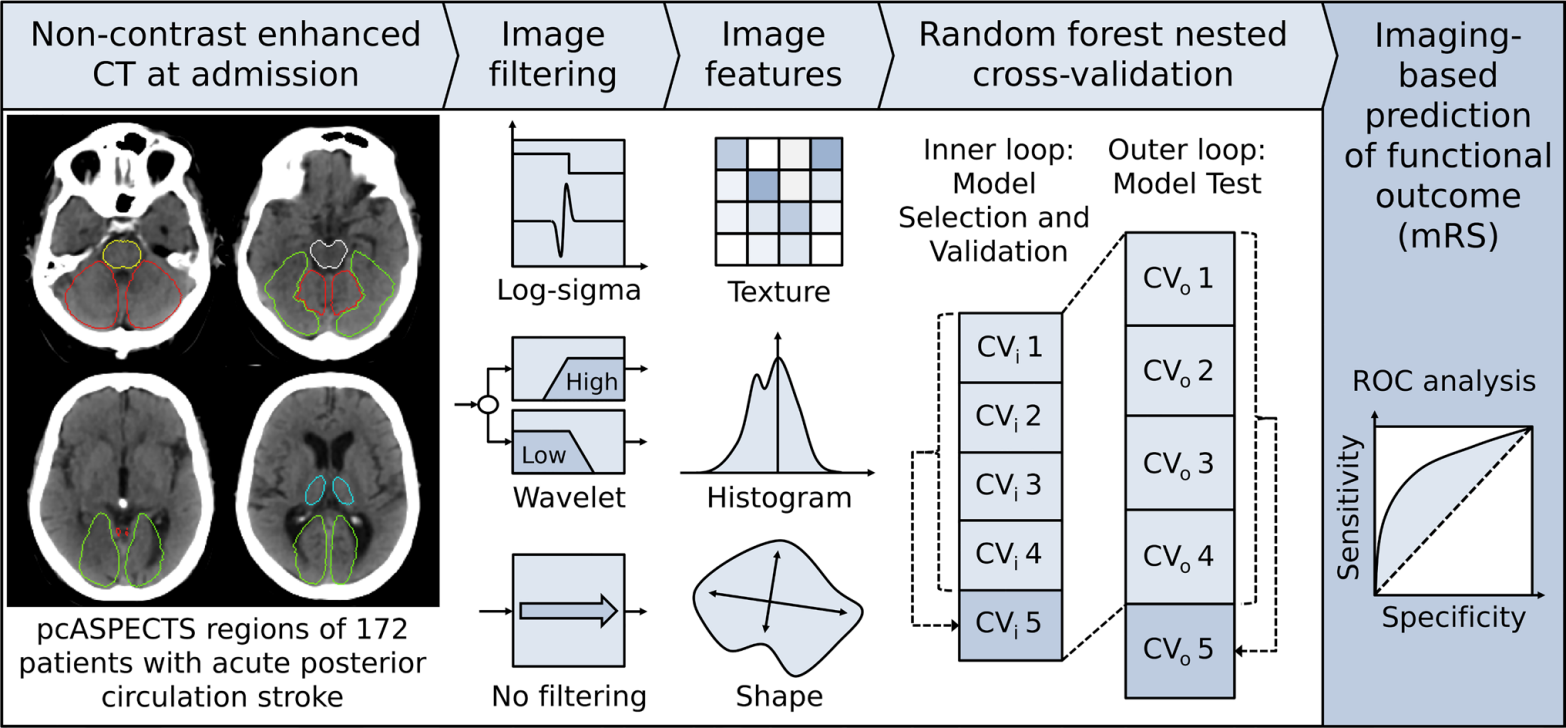
Schematic overview of proposed imaging-based outcome prediction pipeline. *CV* cross validation set, *mRS* modified Rankin Scale, *pc-ASPECTS* posterior circulation Acute Stroke Prognosis Early CT Score, *ROC* receiver-operating-characteristic

A graphical flow chart of the proposed ML-based algorithm for prediction of the clinical outcome is depicted in Fig. 1.

## Results

Our analysis included NCCT images of 1376 pc-ASPECTS regions extracted from 172 patients (77 females, median age 74 years, interquartile range (IQR): 61–79 years) with acute stroke in the posterior circulation. NIHSS assessments were available for 149 patients. Median NIHSS score at admission was 15 (IQR 5–42), 94 patients (54.7%) underwent successful recanalization with TICI (thrombolysis in cerebral infarctions) score ≥ 2b, 79 patients (45.9%) were treated with intravenous thrombolysis (Table 1). 57 patients (33.1%) reached a favorable outcome of mRS ≤ 2 at day 90.

**Table 1:** Patient characteristics

	Total number of patients *n* = 172	mRS at 90 days ≤ 2 *n* = 57 (33%)	mRS at 90 days > 2 *n* = 115 (67%)	*p* value
Age in years, median (IQR)	74 (61–79)	72 (60–78)	74 (62–80)	0.271
Female sex, *n* (%)	77 (44.77%)	19 (33.33%)	58 (50.43%)	0.031
Hypertension, *n* (%)	122 (70.93%)	36 (63.16%)	86 (74.78%)	0.218
Diabetes mellitus, *n* (%)	44 (25.58%)	13 (22.81%)	31 (26.96%)	0,691
Hyperlipidemia, *n* (%)	46 (26.74%)	18 (31.58%)	28 (24.35%)	0.266
Atrial fibrillation, *n* (%)	62 (36.01%)	22 (38.60%)	40 (34.78%)	0.512
Intravenous thrombolysis, *n* (%)	79 (45.93%)	21 (36.84%)	58 (50.43%)	0.064
Mechanical thrombectomy mTICI 2b/3, *n* (%)	94 (54.65%)	27 (47.37%)	67 (58.26%)	0.001
*A*dmission NIHSS, median (IQR) *n* = 23 missings	15 (5–42)	3 (2–10)	30 (13–42)	< 0.001
NIHSS at 24 h, median (IQR) *n* = 23 missings	6 (2–42)	2 (1–4)	36 (15–42)	< 0.001
Discharge NIHSS, median (IQR) *n* = 23 missings	3 (2–17)	2 (1–3)	17 (10–29)	< 0.001
mRS at 90 days, median (IQR)	4 (1–6)	1 (0–1)	5 (4–6)	< 0.001
pc-ASPECTS, median (IQR)	9 (8–10)	9 (9–10)	9 (7–9)	< 0.001
TICI, median (IQR)	3 (2–3)	3 (2–3)	2 (2–3)	< 0.001
Time from onset to imaging in hours, median (IQR)	4 (1.45–10)	6 (1.75–19)	3.20 (1.40–6)	0.024

Demographic and clinical characteristics of the study cohort

*IQR* interquartile range, *mRS* modified Rankin Scale, *NIHSS* National Institute of Health Stroke Scale, *pc-ASPECTS* posterior circulation Acute Stroke Prognosis Early CT Score, *TICI* thrombolysis in cerebral infarctions

### Logistic regression analysis

Logistic regression for mRS ≤ 2 (good outcome) of the conventional predictors (conventional pc-ASPECTS, NIHSS at admission and age) on the entire dataset showed significant coefficients for pc-ASPECTS and NIHSS at admission (P-value < 0.05), age was not significantly associated with good outcome (Table 2). Optimal cut-off values (Youden’s index) indicate that patients with pc-ASPECTS ≥ 8 have a significantly higher probability to achieve mRS ≤ 2 with odds ratio of 11.07 (95% CI [2.55; 48.02]). Also patients with NIHSS at admission < 10 have a significantly higher chance of good outcome with odds ratio of 16.17 (95% CI [7.01; 37.32]).

**Table 2:** Logistic regression of conventional predictors for mRS90 ≤ 2 (good outcome)

	Regression coefficient	Standard error	P-value	Odds ratio	95% CI lower	95% CI upper
pc-ASPECTS	0.48	0.14	< 0.001	1.62	1.23	2.14
Intercept	− 4.87	1.27	< 0.001	0.01		
pc-ASPECTS ≥ 8	2.40	0.75	< 0.001	11.07	2.55	48.02
Intercept	− 2.80	0.73	< 0.001	0.06		
NIHSS at admission	− 0.10	0.02	< 0.001	0.90	0.87	0.94
Intercept	1.07	0.31	< 0.001	2.91		
NIHSS at admission < 10	2.78	0.43	< 0.001	16.17	7.01	37.32
Intercept	− 1.71	0.28	< 0.001	0.18		
Age	− 0.01	0.01	0.38	0.99	0.97	1.01
Intercept	− 0.04	0.77	0.96	0.96		

Logistic regression of conventional clinical information for mRS 90 days ≤ 2 (good outcome). Optimal cut-off values were determined using Receiver-Operating-Characteristic curve analysis Youden’s index. Results are based on 149 patients from 2 different centers.

*mRS* modified Rankin Scale, *NIHSS* National Institute of Health Stroke Scale, *pc-ASPECTS* posterior circulation Acute Stroke Prognosis Early CT Score

### Predictive models for functional outcome

**Fig. 2:**
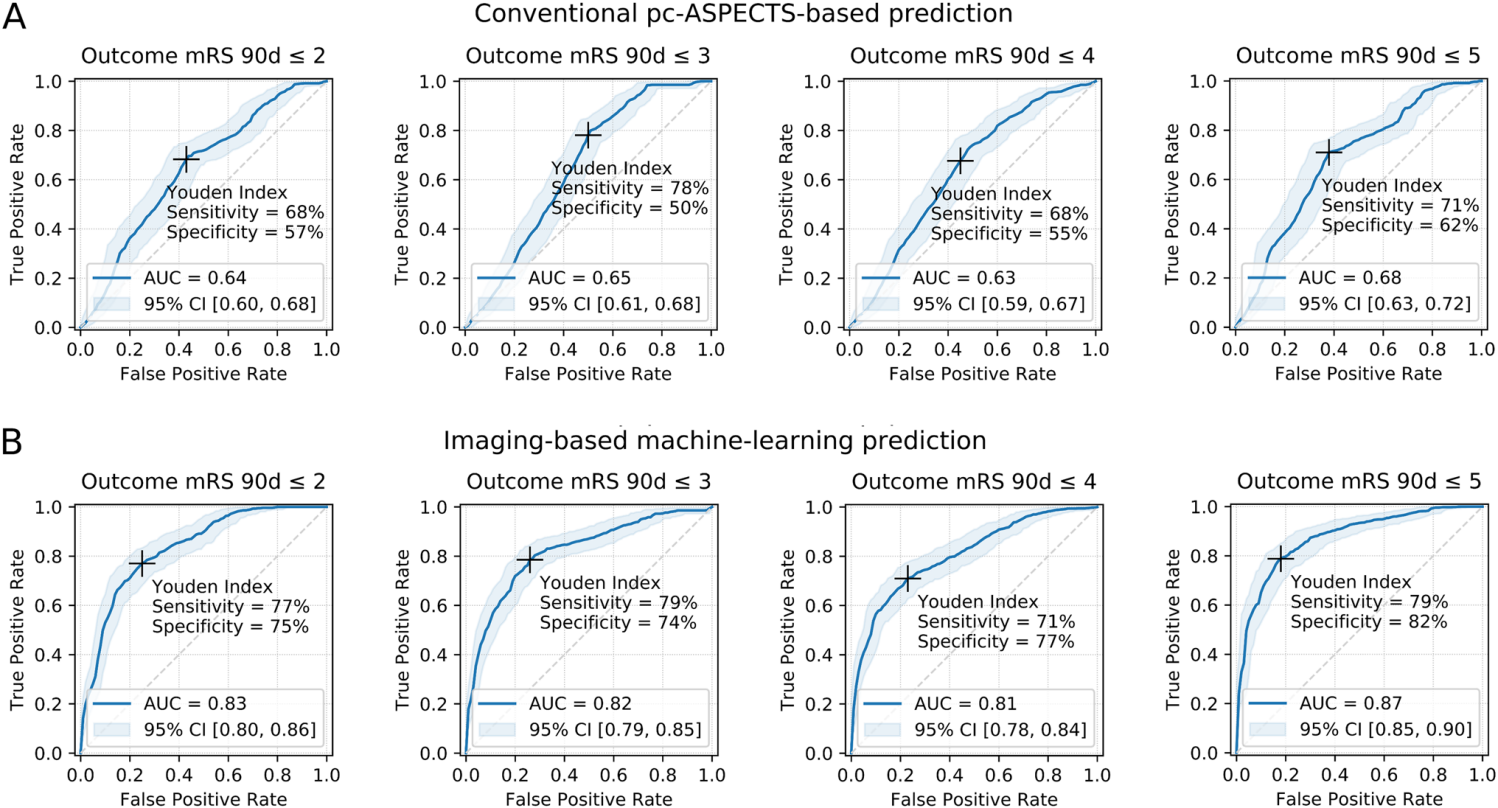
Imaging-based prediction of outcome in patients with posterior circulation stroke at admission. ROC curves, AUCs and maximum Youden index at different mRS cut-off values for respective binary classification tasks. **A** Univariate logistic regression models employing conventional pc-ASPECTS ratings; **B** pure imaging-based random forest machine learning algorithms. Results are based on nested 5-fold cross validation of 172 patients from 2 different centers. Bonferroni corrections have been applied to account for alpha spending error. *CI* confidence interval, *d* days, *mRS* modified Rankin Scale, *pc-ASPECTS* posterior circulation Acute Stroke Prognosis Early CT Score, *ROC AUC* receiver-operating-characteristic area-under-the-curve

**Fig. 3:**
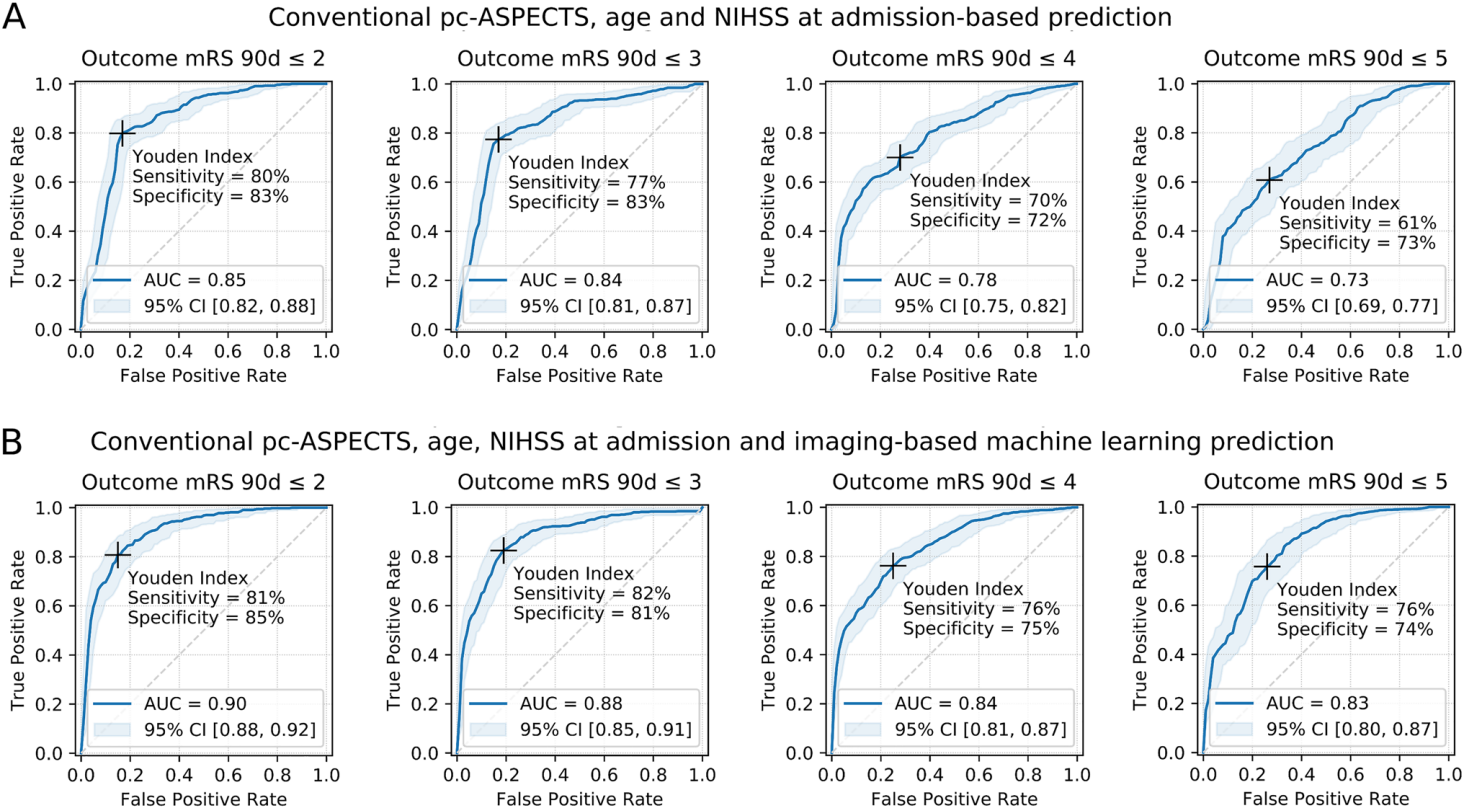
Imaging and clinical data-based prediction of outcome in patients with posterior circulation stroke at admission. ROC curves, AUCs and maximum Youden index at different mRS cut-off values for respective binary classification tasks. **A** Multivariate logistic regression models employing conventional predictors of outcome (conventional pc-ASPECTS ratings, NIHSS at admission, age) and **B** Combined models utilizing information derived from conventional predictors and machine learning-based image analysis. Bonferroni corrections have been applied to account for alpha spending error. Results are based on nested five-fold cross validation of 149 patients from two different centers. *CI* confidence interval, *d* days, *mRS* modified Rankin Scale, *NIHSS* National Institutes of Health Stroke Scale, *pc-ASPECTS* posterior circulation Acute Stroke Prognosis Early CT Score, *ROC AUC* receiver-operating-characteristic area under the curve

**Fig. 4:**
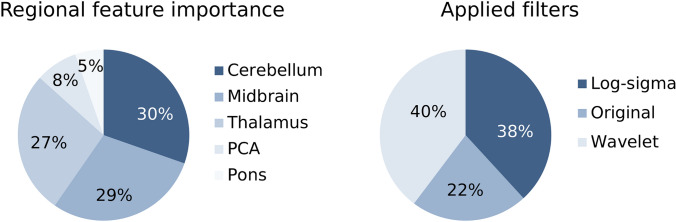
Predictive value of quantitative image features. Pie charts show regional distribution of features and applied filters in utilized top-300 predictors. Results are based on nested five-fold cross validation of 172 patients from two different centers. PCA: Posterior cerebral artery

Areas under the receiver operating characteristic curves (ROC AUCs) of the test sets using conventionally rated pc-ASPECTS in an univariate logistic regression reached 0.63 (95% CI [0.59; 0.67]) for mRS ≤ 4 to 0.68 (95% CI [0.63; 0.72]) for mRS ≤ 5. Pure imaging-based machine learning classifier ROC AUCs were lowest for mRS ≤ 4 with 0.81 (95% CI [0.78; 0.84]) and highest for mRS ≤ 5 with 0.87 (95% CI [0.85; 0.90]). Employing multidimensional conventional predictors (conventional pc-ASPECTS, NIHSS at admission and age) yielded ROC AUCs of 0.73 (95% CI [0.69; 0.77]) for mRS ≤ 5 (lowest) to 0.85 (95% CI [0.82; 0.88]) for mRS ≤ 2. Overall, highest predictive performance was observed for the combined clinical data and machine learning-based model with ROC AUCs of 0.83 (95% CI [0.80; 0.87]) for mRS ≤ 5 (lowest) to 0.90 (95% CI [0.88; 0.92]) for mRS ≤ 2 (highest) (Figs. 2 and 3 and Table 3). Results show that the predictive performance of machine learning-based evaluation of quantitative image features was higher compared to the predictive value of conventional pc-ASPECTS metrics (*p* values < 0.05). If combined with additional clinical data (NIHSS at admission and age), the conventional prediction model achieved slightly better metrics for differentiating lower mRS values (≤ 2 and ≤ 3), however, these differences were not significant. For the mRS ≤ 4 and ≤ 5 classification tasks, quantitative image features-based algorithms reached higher performance with significant differences in all metrics for mRS ≤ 5 (survival). The integrated model employing information from conventional pc-ASPECTS ratings, clinical data and machine learning-based evaluation of quantitative image features showed superior results versus conventional pc-ASPECTS and clinical data by trend for all mRS cut-offs. Improvements were observed for ROC AUC in mRS ≤ 2 prediction (ROC AUC = 0.90 vs. 0.85, *p* value < 0.05) and for all metrics in mRS ≤ 5 prediction (ROC AUC = 0.83 vs. 0.73, *p* value < 0.05). Feature importance analyses of the mean top 300 predictors of all training data sets show that pc-ASPECTS regions with the highest predictive power are cerebellum (30%), midbrain (29%) and thalamus (27%). The largest share of predictive value was mainly derived from wavelet (40%) and log-sigma (38%) filtered images. Unfiltered original images contributed 22% to total predictive power (Fig. 4). Within feature classes, texture metrics and first order statistics were used at equal proportions.

**Table 3:** Classification performance of imaging-based outcome prediction

Prediction mRS	Patients (n; %)	Classifier model	ROC AUC [95% CI]	MCC maximum [95% CI]	Youden index [95% CI]	Accuracy [95% CI]
mRS ≤ 2	57/172 (33%)	pc-ASPECTS log reg	0.64 [0.60; 0.68]	0.21 [0.18; 0.24]	26% [18%; 33%]	65% [62%; 68%]
	57/172 (33%)	Img-based ML	0.83 [0.80; 0.86]	0.52 [0.47; 0.58]	52% [46%; 58%]	76% [73%; 79%]
	53/149 (31%)	pc-ASPECTS and clin. data log reg	0.85 [0.82; 0.88]	0.62 [0.57; 0.66]	63% [57%; 69%]	82% [79%; 85%]
	53/149 (31%)	Img-based and clin. data ML	0.90* [0.88; 0.92]	0.65 [0.60; 0.69]	66% [60%; 72%]	83% [81%; 86%]
mRS ≤ 3	69/172 (40%)	pc-ASPECTS log reg	0.65 [0.61; 0.68]	0.36 [0.23; 0.30]	29% [23%; 35%]	62% [58%; 65%]
	69/172 (40%)	Img-based ML	0.82 [0.79; 0.85]	0.52 [0.46; 0.56]	52% [47%; 58%]	76% [73%; 79%]
	65/149 (38%)	pc-ASPECTS and clin. data log reg	0.84 [0.81; 0.87]	0.60 [0.55; 0.65]	60% [54%; 66%]	80% [77%; 83%]
	65/149 (38%)	Img-based and clin. data ML	0.88 [0.85; 0.91]	0.63 [0.59; 0.67]	64% [58%; 69%]	82% [79%; 84%]
mRS ≤ 4	96/172 (56%)	pc-ASPECTS log reg	0.63 [0.59; 0.67]	0.25 [0.18; 0.31]	23% [16%; 29%]	62% [59%; 65%]
	96/172 (56%)	Img-based ML	0.81 [0.78; 0.84]	0.48 [0.42; 0.53]	48% [42%; 54%]	74% [71%; 77%]
	87/149 (51%)	pc-ASPECTS and clin. data log reg	0.78 [0.75; 0.81]	0.41 [0.35; 0.46]	42% [35%; 48%]	71% [67%; 74%]
	87/149 (51%)	Img-based and clin. data ML	0.84* [0.81; 0.87]	0.51* [0.46; 0.56]	51% [45%; 57%]	75% [72%; 78%]
mRS ≤ 5	127/172 (74%)	pc-ASPECTS log reg	0.68 [0.63; 0.72]	0.3 [0.24; 0.36]	33% [26%; 40%]	69% [65%; 72%]
	127/172 (74%)	Img-based ML	0.87† [0.85; 0.90]	0.56† [0.51; 0.60]	61%† [55%; 67%]	80%† [77%; 82%]
	115/149 (67%)	pc-ASPECTS and clin. data log reg	0.73 [0.69; 0.77]	0.28 [0.21; 0.35]	34% [26%; 41%]	64% [60%; 67%]
	115/149 (67%)	Img-based and clin. data ML	0.83* [0.80; 0.87]	0.49* [0.43; 0.54]	50%* [42%; 57%]	75%* [72%; 78%]

Prediction of outcome in patients with posterior circulation stroke at admission: mRS cut-off values for classification tasks, number of patients with respective outcome (positive class) and performance metrics of logistic regression models employing conventional pc-ASPETCS ratings, pure imaging-based machine learning algorithms, multivariate logistic regression models employing conventional predictors of outcome (conventional pc-ASPECTS ratings, NIHSS at admission, age) and combined models utilizing information derived from conventional predictors and machine learning-based image analysis. Metrics are shown at Youden index maximum cut-off points. Results are based on nested fivefold cross validation of 172 (149) patients from two different centers. Bonferroni corrections have been applied to account for alpha spending error

*CI* confidence interval, *MCC* Matthews correlation coefficient, *mRS* modified Rankin Scale, *pc-ASPECTS* posterior circulation Acute Stroke Prognosis Early CT Score, *ROC AUC* receiver-operating-characteristic area under the curve

*p value combined model vs. pc-ASPECTS and clinical data model < 0.05

^†^*p* value imaging-based machine learning vs. pc-ASPECTS and clinical data model < 0.05

## Discussion

In this study, we developed a machine learning approach for predicting functional outcome of patients with posterior circulation stroke based on multidimensional quantitative image analysis of pc-ASPECTS regions in admission NCCTs and basic clinical data available at admission. The study is based on a cohort of 172 patients, of which 57 (33.1%) achieved a favorable outcome of mRS ≤ 2 at day 90. This corresponds to the results of the BASIC trial with a recently reported total share of 32.7% for mRS ≤ 2 at day 90 (35.1% in the intervention arm vs. 30.1% in the control arm) in patients with basilar artery occlusions [32].

Conventional logistic regression and cut-off point optimization confirmed that high pc-ASPECTS (optimal cut-off at pc-ASPECTS ≥ 8) and low NIHSS at admission (optimal cut-off at NIHSS < 10) are significant and independent predictors of good outcome. These results are in line with the findings of other studies [2, 12].

The proposed ML-approach employing quantitative image features provided high discriminatory accuracy between good and poor functional outcome at different mRS thresholds; observed performance metrics were superior or equal to conventional clinical and imaging-based assessments. For predicting mRS ≤ 2, ROC AUC, sensitivity and specificity were 0.90, 81% and 85% for the integrated machine learning classifier; 0.85, 80% and 83% for conventional pc-ASPECTS with clinical data and 0.64, 68% and 57% for conventional pc-ASPECTS alone. Our analysis also showed that employing multidimensional clinical predictors (conventional pc-ASPECTS, NIHSS at admission and age) improved accuracy with a statistically significant increase in ROC AUC compared to using conventional pc-ASPECTS alone. However, even the solely imaging-based machine learning approach achieved higher discriminatory power than conventional pc-ASPECTS.

Earlier studies have investigated the predictive power of conventional pc-ASPECTS ratings based on different imaging modalities and clinical parameters [2, 16, 19, 21, 30]. Lin et al. [16] report ROC AUC for pc-ASPECTS and NIHSS at admission of 0.69 and of 0.77 if both parameters are combined. Other works focused on outcome prediction after endovascular therapy and show ROC AUC of 0.74 for NIHSS at admission and 0.72 for pc-ASPECTS [21]. Based on CT perfusion imaging parameters, pc-ASPECTS ROC AUC was reported to achieve 0.64 (mean transit time) to 0.82 (cerebral blood volume) [2]. pc-ASPECTS based on DWI was shown to be a predictor of clinical outcome with ROC AUC of 0.82 [19]. To date, all published studies employ conventional regression analysis. None of the published analyses investigated the discriminatory power of ML-based quantitative image assessment in a train, validation and test approach.

Our study has the following limitations: first, generalizability might be limited due to the retrospective nature with inherent selection bias and its relatively small sample size. An expansion of sample size in a prospective study design would certainly contribute to further improving generalizability of results. However, low variability of results across different validation sets suggests sufficient robustness for assessing general feasibility and limitations of the approach. Second, differences in recanalization treatment possibly have influenced patients´ outcome. However, our results were confirmed throughout the whole patient collective despite different recanalization results even though our approach did include only variables available at admission. This observation indicates that clinical and imaging data at admission might already include information regarding probabilities of specific treatment strategies (e.g., decision for mechanical thrombectomy based on age, NIHSS, pc-ASPECTS). Furthermore, our findings are supported by the results of the BASICS trial that did not report a significant difference in functional outcome for patients treated with endovascular therapy plus best medical management vs. best medical management alone [32]. Third, limitations typically associated with quantitative radiomics-based image analysis and classification may compromise generalizability of the results [1, 5, 6, 11, 13]. These limitations include differences in image acquisition settings, for example size of the field of view or gantry tilt, and under- or overfitting of machine learning algorithms. Bias of these factors was minimized through employment of standardized NCCT scans and the application of Random Forest algorithms that are comparably stable with regards to overfitting. The risk of overfitting was also reduced by evaluating multiple different models in a nested cross-validation approach. Due to standardized and calibrated quantitative imaging parameters and signal intensity processing of CT scanners we assume neglectable bias on classifier performance in a generalized setting. Fourth, with NCCT being the most widely performed brain-imaging technique in acute pc stroke settings [9], our analysis did not integrate CT angiography, CT perfusion or MR imaging. An extension to these imaging modalities could have further improved the results [22, 26–28] as NCCT images only offer limited sensitivity for detecting ischemia compared to e.g. diffusion-weighted imaging [34]. However, both scores—conventional ASPECTS (Alberta Stroke Program Early CT Score) for anterior circulation and pc-ASPECTS—are originally based on evaluations of acute NCCT scans. NCCT scans at admission are fast and the technique is available in most hospitals. Furthermore, NCCT imaging is a fundamental part of most standard-of-care stroke protocols. Fifth, the acquisition resolution of NCCT scans was limited to < 5 mm in slice thickness. The utilization of higher resolution images could improve classification performance. Sixth, the manual definition of pc-ASPECTS areas still implies a certain degree of observer-dependence within the machine learning process. To minimize its influence, we derived standard maps from delineations obtained from 63 healthy subjects. Further, it was shown that radiomic features are comparably stable with regards to variations in segmentations [23, 37].

### Conclusion

We developed a machine-learning based classifier that predicts functional outcome of acute posterior circulation stroke patients based on quantitative multidimensional analysis of pc-ASPECTS regions. We observed higher classification performance metrics than achieved in conventional clinical and imaging-based assessments. The proposed algorithm might therefore (1) allow for an early prognosis of the patient’s long-term functional status (2) optimize triage for additional MR imaging diagnostics and allocation of best possible medical care [36] and (3) could facilitate required arrangements of the patient’s social environment at an early point of time.
